# Molecular Dynamics of CYFIP2 Protein and Its R87C Variant Related to Early Infantile Epileptic Encephalopathy

**DOI:** 10.3390/ijms23158708

**Published:** 2022-08-05

**Authors:** Ísis V. Biembengut, Patrícia Shigunov, Natalia F. Frota, Marcos R. Lourenzoni, Tatiana A. C. B. de Souza

**Affiliations:** 1 Laboratory for Structural and Computational Proteomics, Carlos Chagas Institute, Fundação Oswaldo Cruz Paraná (Fiocruz-PR), Curitiba 80320-290, Brazil; 2 Laboratory of Basic Biology of Stem Cells, Carlos Chagas Institute, Fundação Oswaldo Cruz Paraná (Fiocruz-PR), Curitiba 80320-290, Brazil; 3 Campus do Pici (Bloco 873), Federal University of Ceara (UFC), Fortaleza 60440-970, Brazil; 4 Research Group on Protein Engineering and Health Solutions (GEPeSS), Fundação Oswaldo Cruz Ceará (Fiocruz-CE), São José, Precabura, Eusébio 61773-270, Brazil

**Keywords:** CYFIP2, early infantile epileptic encephalopathies, WRC complex, molecular dynamics

## Abstract

The CYFIP2 protein (cytoplasmic FMR1-interacting protein 2) is part of the WAVE regulatory complex (WRC). CYFIP2 was recently correlated to neurological disorders by the association of the R87C variant with early infantile epileptic encephalopathy (EIEE) patients. In this set of syndromes, the epileptic spasms and seizures since early childhood lead to impaired neurological development in children. Inside the WRC, the variant residue is at the CYFIP2 and WAVE1 protein interface. Thus, the hypothesis is that the R87C modification weakens this interaction, allowing the WRC complex’s constant activation. This work aimed to investigate the impacts of the mutation on the structure of the WRC complex through molecular dynamics simulation. For that, we constructed WRC models containing WAVE1-NCKAP1 proteins complexed with WT or R87C CYFIP2. Our simulations showed a flexibilization of the loop comprising residues 80–110 due to the loss of contacts between internal residues in the R87C CYFIP2 as well as the key role of residues R/C87, E624, and E689 in structural modification. These data could explain the mechanism by which the mutation impairs the stability and proper regulation of the WRC.

## 1. Introduction

Early infantile epileptic encephalopathies (EIEE) are a rare group of neurodevelopment syndromes that appear in early childhood. In this pathology, convulsions and epileptic episodes compromise the proper neural development [1,2]. The precise incidence of this set of syndromes is not reported. However, it is estimated that infantile spasm syndromes (or West syndrome), which are also aggregated within EIEE, have an occurrence of about 0.25 cases per 1000 live births. Their prevalence is around 1 in 10,000 children aged 10 years [3,4,5].

With the recent advances in next-generation sequencing (NGS), single mutations in diverse genes have already been correlated with EIEE, such as CYFIP2 [6,7]. The CYFIP2 protein participates in the WAVE regulatory complex (WRC), which is involved in the regulation of actin filament polymerization, through an interaction with the Arp2/3 complex [8]. The WRC complex is a pentameric complex, which has 36 possible combinations due to the participation of homologous proteins, formed by the proteins WAVE1 (or WAVE2 or WAVE3), CYFIP2 (or CYFIP1), NCKAP1 (or NCKAP1L), ABI1 (or ABI2 or ABI3), and BRK1 [9,10,11,12]. The three-dimensional structure of the WRC complex was solved by X-ray crystallography (PDB 3P8C and PDB 4N78), having in its composition the WAVE1, CYFIP1, NCKAP1, ABI2, and BRK1 proteins [13].

Recently, de novo mutations in residue R87 of CYFIP2 were associated with EIEE [7]. After this initial report, other studies reported EIEE patients with mutations in the same residue (the R87C substitution was the most common), with at least 10 patients reported until 2020 [14,15,16,17]. Through the analysis of the structure of the WRC complex, it was hypothesized that the variants could compromise the interaction of CYFIP2 with WAVE, thus promoting a constant and unregulated activation of the complex and consequently deregulating actin filament polymerization [7,17]. This hypothesis was supported by a study where lamellipodia formation was used to assess the impact of the mutation. There, the CYFIP2 R87C expression in CYFIP1-2 knockout cells showed the recovery of lamellipodia, even in the absence of WRC complex activation by RAC1. This result suggested the constant activation of the WRC in the presence of mutated CYFIP2 [18]. This whole scenario would compromise the structures of the neuronal dendritic spines, which could be the cause of the pathology in the patients [19]. Moreover, CYFIP2 R87 variants may impact the formation of stress granules. Lee and colleagues showed that cells with CYFIP2 R87 variants form clusters containing CYFIP2 and that the CYFIP2 interactome contains 23 proteins that are common components of stress granules. In addition to its effects on WRC regulation, CYFIP2 clustering could also impair its function [20]. However, neither of these studies described the exact mechanism by which the mutation compromises the structure of CYFIP2 and the WRC complex.

Here, we were interested in investigating how these variants affect the structure and dynamics of CYFIP2. We therefore conducted molecular dynamics simulations of CYFIP2 within its WRC interactors and analyzed the effect of the mutations on the dynamic properties of CYFIP2. Our simulations establish a flexibilization in the loop 80–110 in the CYFIP2 R87C variant that could impair its own stability and interactions with WAVE protein. Furthermore, our results identified that the loss of contacts between residues R87, E624, and E689 could explain these structural modifications. Based on our models, these structural modifications might play an essential role in the stability and proper regulation of the WRC complex and, therefore, could help to explain these mutants’ phenotype. Moreover, our findings may provide information for future studies interested in the structure and dynamics of WRC complex mutants and possible ways to restore proper CYFIP2 function.

## 2. Results

### 2.1. CYFIP2 WT and CYFIP2 R87C Evolution through the Simulations

**Figure 1:**
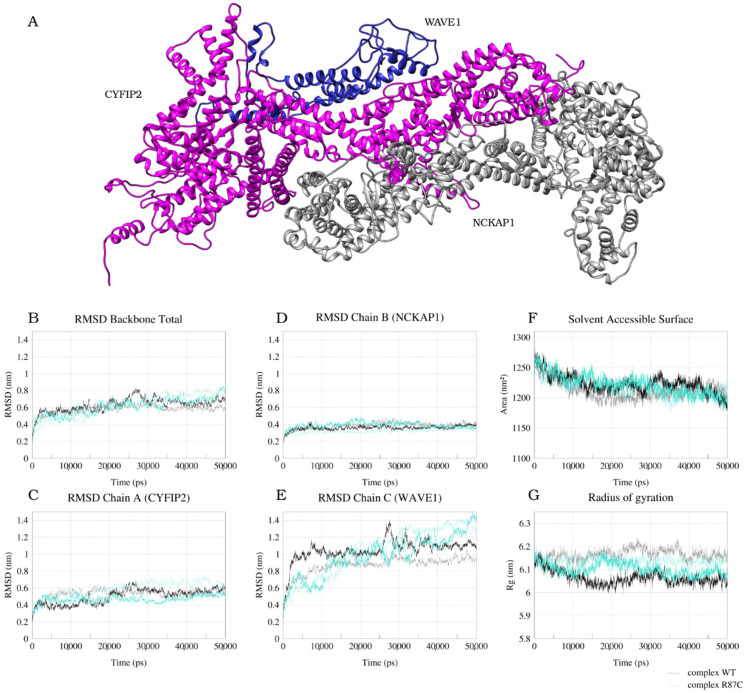
Structure and conformational evolution through the entire MD simulation of WT and R87C complex models. Ribbon representation of CYFIP2 (magenta), NCKAP1 (gray), and WAVE1 (blue) model (**A**). Plot of root-mean-square deviation (RMSD) calculated using the original model as a reference for the backbone of all chains (**B**), CYFIP2 chain (**C**), NCKAP1 chain (**D**), and WAVE1 chain (**E**). Plot of solvent-accessible surface area (SASA) (**F**). Plot of the radius of gyration (Rg) (**G**). Data for complex WT simulation are represented by dark (first replica) and light (second replica) gray lines. Data for complex R87C simulation are represented by dark (first replica) and light (second replica) cyan lines.

The crystallographic structure of the WRC complex [13] shows a pentameric complex composed of CYFIP1, NCKAP1, WAVE1, Abi2, and BRK1 proteins. The CYFIP2 protein (CYFIP1 homolog) is also part of the complex, and here it was our object of study, together with the R87C variant related to EIEE. Therefore, we constructed homology models of CYFIP2 and CYFIP2 R87C and reconstructed the complex, generating models with the replacement of the CYFIP1 protein by CYFIP2 (here named complex WT) or CYFIP2 R87C (here named complex R87C). The structures of Abi2 and BRK1 were excluded from the complex models as a simplification (considering the computational cost) because its unstructured regions could not be modeled by homology within the complex. These proteins do not interact with the region within the R87C mutation in CYFIP2 but could affect the region allosterically. We accounted for this simplification in our analysis, always comparing the results obtained for the complex simulation with the isolated CYFIP2 models. In our models, CYFIP2 assumes a planar structure alone and within the complex, as does CYFIP1 in the crystallographic structure. It is also possible to identify three distinct domains of CYFIP2: the first (N-terminal) with a more globular structure, where residue 87 is located, the second (central) with a more planar structure, and the third (C-terminal), smaller and with a globular configuration (Figure 1A). Comparing our initial models with the crystallographic structure, we achieved similar structures with a RMSD of 0.848 Å between all atom pairs of the initial complex WT model and the crystallographic structure (3P8C), calculated using Chimera. All models obtained acceptable scores under a Molprobity geometric evaluation (Table S1).

The backbone RMSDs from the initial models of the WT and R87C complex are displayed in Figure 1. Both WT and R87C models moved away from the starting structure during the first 5 ns of the simulation, reaching final RMSDs values between 0.6 and 0.8 nm after 20 ns that remained through the rest of the simulation (Figure 1B). The convergence of RMSDs indicates a convergence of the trajectory in our simulation time. When we performed the analysis for each chain of the system separately, chains A (CYFIP2) and B (NCKAP1) reached an equilibrium in less than 5 ns between 0.4 and 0.6 nm (Figure 1C,D). Meanwhile, the chain C (WAVE1) deviated more from the initial structure, with RMSD values converging between 1 and 1.1 nm for the WT complex after 10 ns and between 1.2 and 1.4 nm for the R87C complex after almost 30 ns of simulation (Figure 1E). Looking into the trajectory, this more significant deviation of the WAVE1 chain can be explained by the movement of its N-terminal helix. This was expected, considering that in our models, the helix is not stabilized by the presence of Abi2 and BRK1 as in the crystallographic structure. This significant movement in the WAVE1 N-terminal helix can also be seen in the RMSF graph (Figure S2), where the residue fluctuation in this region reaches values of 2 nm.

Regarding the simulation of isolated CYFIP2 models, the RMSDs show that both the WT and R87C models reached equilibrium around 30 ns but with a higher RMSD for the WT model, which was between 0.7 and 0.9 nm. This implies that the CYFIP2 WT final structure differed more from the initial model than CYFIP2 R87C (Figure S1B). Both structures reached a convergence of the trajectory but with some conformational differences, as shown in Figure S1A.

The radius of gyration (Rg) ranged between 6 and 6.2 nm during the entire simulation, considering both replicas and both WT and R87C complexes (Figure 1G). This indicates that there was no major conformation reorganization during our entire simulation. The same was observed in both the CYFIP2 WT and R87C simulations but with a lower Rg range, as expected, between 5.15 and 5.35 nm (Figure S2D). All models showed a tendency to decrease the surface accessible to the solvent (SASA) in the first 15 ns of simulation. The SASA values decreased from an average of 1250 nm^2^ to a running average of about 1200 nm^2^ for the complexes (Figure 1F) and from ~680 nm^2^ to 640 nm^2^ for the isolated CYFIP2 WT and R87C models (Figure S2C). This may indicate a better accommodation of the interactions compared to the initial WT and R87C models before the simulation. These analyses indicate that in 50 ns of simulation, we achieved convergence for all complexes, and we achieved convergence in 100 ns for all the isolated CYFIP2 models.

### 2.2. The Simulation Showed A Flexibilization of the Loop (80–110) in Complex R87C

**Figure 2:**
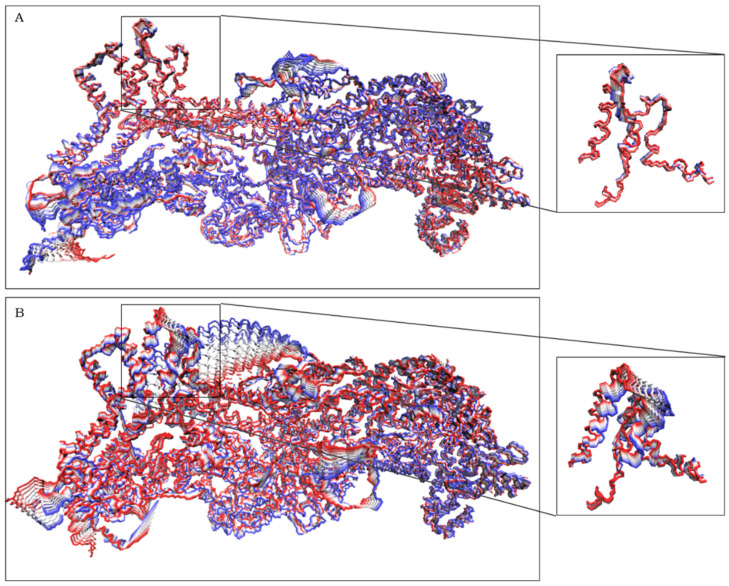
Projections of the trajectories from both replicas over the first vector for complex WT (**A**) and complex R87C (**B**). On the right are highlighted residues 80–120 from CYFIP2 and 131–161 from WAVE1. Ten frames per trajectory are shown from the highest positive (red) to the highest negative (blue) eigenvalues.

In order to analyze the most relevant movements during the simulations, we performed a principal component analysis (PCA) for the complexes. In PCA, the samples are rewritten on the basis of the eigenvectors. The components of the formed vector are the principal components, and their variances are the eigenvalues [21], with the eigenproblem playing an essential role in molecular alignment [22]. This analysis decomposes the movements during the simulation, aiming to differentiate between divergent behaviors and small fluctuations. When we projected the trajectory of the complexes on the first vector (corresponding to 30% of the total variation for the WT complex and 50%/45% for the R87C complex—Figure S2), we observed a great difference in behavior between the WT complex and the R87C complex (Figure 2). This projection along the first vector showed a large distortion in the loop region to which residue 87 belongs only in the variant complex. This indicates the possibility of greater flexibility of this region due to the mutation. This flexibilization could lead to instability of the region in the CYFIP2 protein and the complex as a whole.

**Figure 3:**
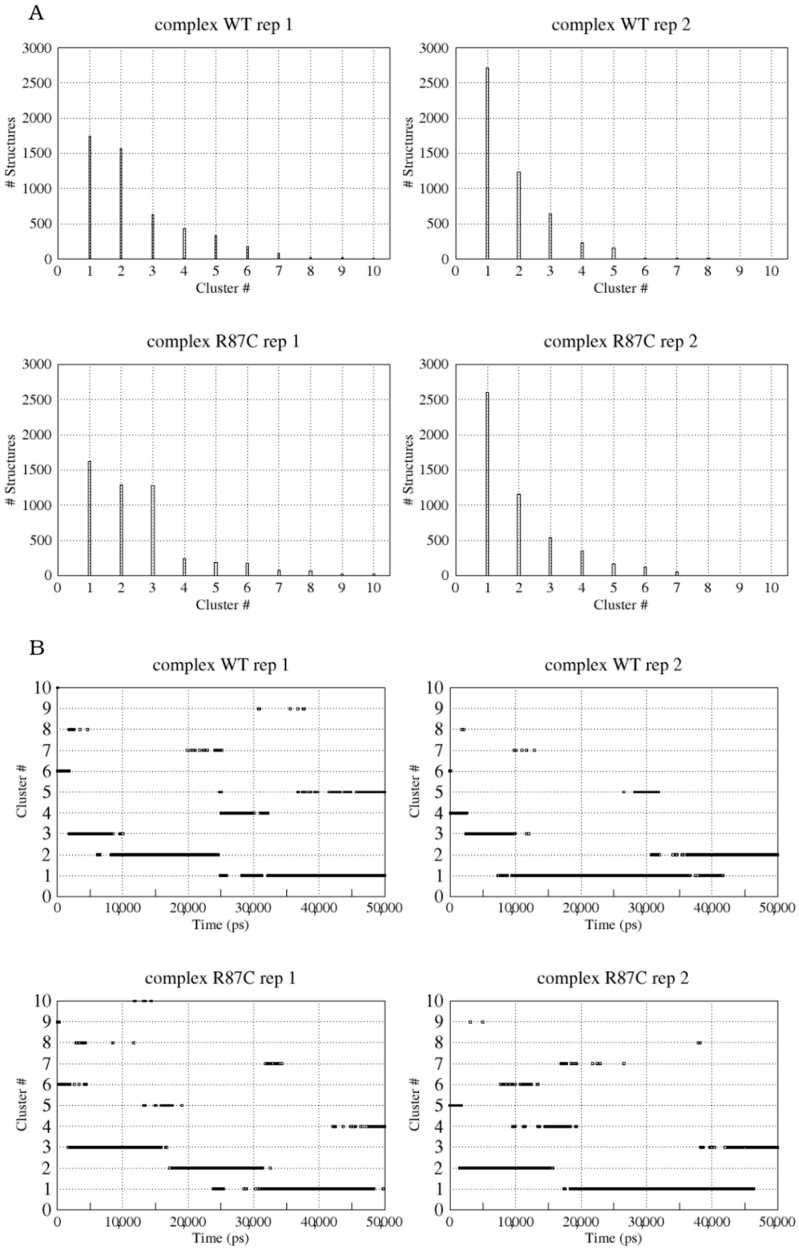
Cluster grouping of structures for first and second replicas of the WT and R87C complex MD simulations. The number of frames grouped in each cluster (**A**). Time evolution of each cluster (**B**). A threshold value of 0.3 nm was used for clustering.

**Figure 4:**
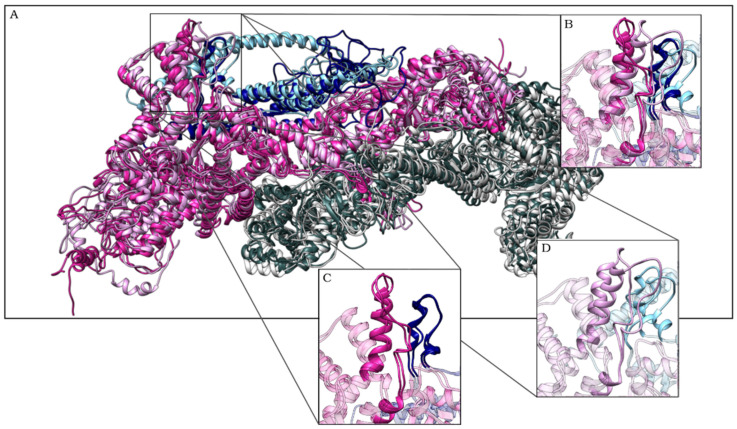
Superimposed structures of the first cluster centroid for both replicas from the WT complex (darker colors) and the R87C complex (lighter colors) (**A**). Representation of CYFIP2 in magenta, NCKAP1 in gray, and WAVE1 in blue. Detail of residues 80–120 from CYFIP2 and residues 131–151 from WAVE1. Superimposed models (**B**), only WT models (**C**), and only R87C models (**D**). Note how this loop diverges for the R87C variant compared to the WT.

To better analyze this change, we clustered the conformations explored by the complexes during the trajectory according to the RMSDs among their Cα atoms. A total of 5000 structures were divided into a maximum of 13 clusters, or families of structures, for the WT complex and 11 clusters for the R87C complex (Figure 3A—data shown only for the first 10 clusters). The clusterization was used to obtain representative structures of the simulation that could be analyzed in more detail. The structure that represents the centroid of the most populous cluster in each simulation was chosen for comparison. These structures are representative of the 25,000 ps to 50,000 ps of simulation for the WT complex (10,000 ps to 40,000 ps in replicate 2) and from 25,000 ps to 50,000 ps for the R87C complex (20,000 ps to 45,000 in replicate 2) (Figure 3B). The superposition of the WT complex and the R87C complex (Figure 4) shows the loop distortion between residues 80 to 110 again.

**Figure 5:**
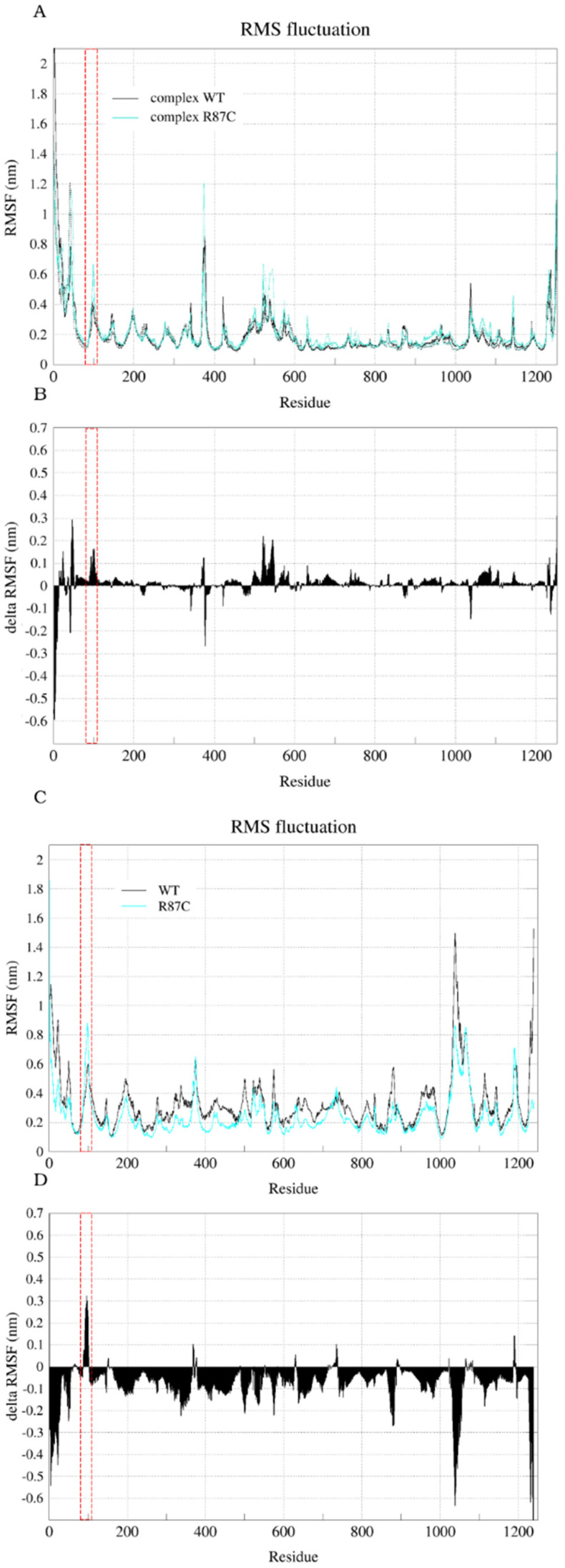
RMSF (calculated with reference to the average structure fitting of C-alphas for the entire structure through the entire trajectory) and delta RMSF for CYFIP2. Root-mean-square fluctuation (RMSF) for each residue ((**A**)—complex and (**C**)—isolated CYFIP2). The delta RMSF values ((**B**)—complex and (**D**)—isolated CYFIP2) were calculated based on the difference in RMSF for each residue (mean of both replicas) in the R87C simulation and the WT simulation. Positive values along the sequence indicate an increase in the flexibility in the mutant. Data for complex WT simulation are represented by dark (first replica) and light (second replica) gray lines. Data for complex R87C simulation are represented by dark (first replica) and light (second replica) cyan lines. Data for the CYFIP2 WT simulation are represented by black lines. Data for the CYFIP2 R87C simulation are represented by cyan lines. Red box highlights the region between residues 80–110 in all graphs.

Finally, in an attempt to assess the flexibility of the R87C model compared to the WT model isolated and in the complex, we calculated the root mean square fluctuation (RMSF) for each CYFIP2 residue throughout the entire simulations (Figure 5A,C). For comparison purposes, we calculated the delta RMSF between the structure of the R87C and the WT models for both isolated (Figure 5B) and within-the-complex (Figure 5D) simulations. For this, we took the RMSF average of each residue between the replicates for each model, and then the difference between the R87C version and the WT version averages was calculated. The regions where negative values predominate indicate a greater rigidity in the R87C version, while the regions where positive values predominate indicate flexibilization. Note that isolated CYFIP2 R87C and the complex R87C presented a flexibilization of its structure around the region between residues 80 and 110. For complex R87C, we also observed a flexibilization of its entire structure, another indication that the mutation could cause a destabilization of the CYFIP2 protein and the WRC complex as a whole, mainly in the 80–110 loop region.

### 2.3. The R87C Mutation Underlies A Flexibilization of Its Loop Inside CYFIP2 Caused by the Loss of Important Interactions

**Figure 6:**
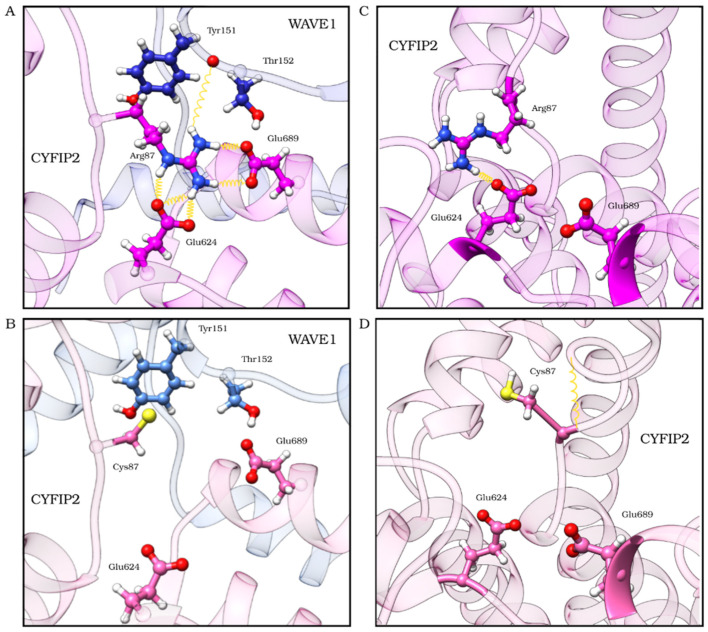
Residues that interact through hydrogen bonds with CYFIP2 R87 within the complex (**A**) and outside the complex (**C**) and how the C87 mutant loses these interactions within the complex (**B**) and outside the complex (**D**), causing the loosening of the loop and resulting in a structural change in the protein during our simulation. CYFIP2 residues are represented in magenta, and WAVE1 residues are represented in blue. Structures of the first cluster centroid are from the first replicate. Yellow springs represent hydrogen bonds.

We hypothesized that this loop flexibilization could be caused by the loss of important interactions at residue 87 due to the mutation. In our initial model (not shown) and in the structure resulting from the complex trajectory clustering, residue 87 forms hydrogen bonds with CYFIP2 residues E624 and E689 and with WAVE1 residue Y151 (Figure 6A). These interactions are lost in the model of the R87C complex (Figure 6B). In the structure resulting from the CYFIP2 trajectory clustering, residue 87 forms hydrogen bonds with CYFIP2 residue E624 (Figure 6C), which is also lost in R87C CYFIP2 (Figure 6D).

We also compared the position of Y151 with residues W86, S88, C89, and F686 from CYFIP2 and P131, P132, P133, L134, and L148 from WAVE1 (Figure S4) because in the inactive WRC complex these residues form a pocket around Y151 [23]. In the R87C complex simulation, we see that the approximation of the WAVE1 chain and the CYFIP2 loop closes this pocket and starts to push Y151 outside of it. This seems to happen because of the loop loosening.

**Figure 7:**
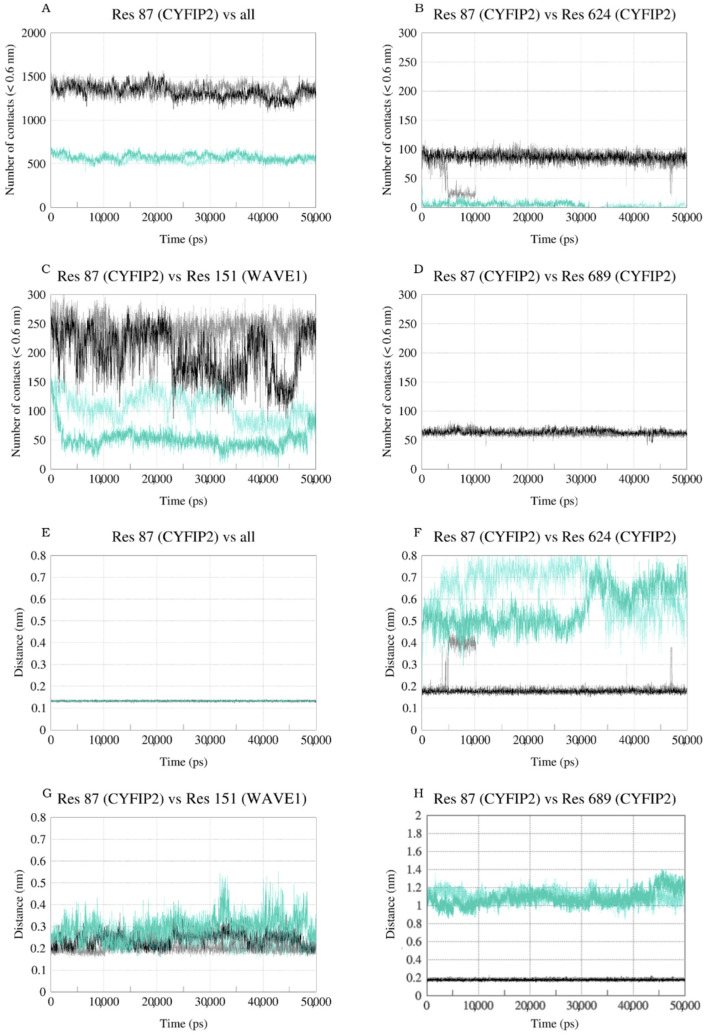
Number of contacts between residue R/C87 and all other residues (**A**), E624 CYFIP2 (**B**), Y151 WAVE1 (**C**), and E689 CYFIP2 (**D**) as a function of simulation time (ps). Minimal distance between residue R/C87 and all other residues (**E**), E624 CYFIP2 (**F**), Y151 WAVE1 (**G**), and E689 CYFIP2 (**H**) as a function of simulation time (ps). Data for complex WT simulation are represented by dark (first replica) and light (second replica) gray lines. Data for complex R87C simulation are represented by dark (first replica) and light (second replica) cyan lines.

We then evaluated the behavior of these interactions throughout the trajectory, looking at the number of contacts between the residues (Figure 7A–D) as well as their minimum distance (Figure 7E–H). The evaluation of the number of contacts was defined with a cutoff of less than 0.6 nm for the distance between the atoms of the two analyzed groups. Residue 87 within the WT complex made about 100 contacts with residue E624 throughout the simulation, with a minimum distance of about 0.2 nm, showing that the interaction between these two residues is stable. Similar behavior existed in the interaction with residue E689, where about 60 contacts were maintained along the trajectory, with a minimum distance of about 0.2 nm. When we looked at these same interactions in the R87C complex, we saw how they were almost entirely lost due to mutation. Less than 10 contacts were observed between residues 87 and E624, with a minimum distance greater than 0.5 nm. There were no contacts between residues 87 and E689, with a minimum distance greater than 1.0 nm throughout the entire simulation. This shows that the absence of contacts in the initial R87C models was maintained during the entire simulation, indicating that no accommodation occurred (in a simulated solvated environment) that could stabilize these interactions and avoid the loop structural destabilization. Moreover, these interactions were stable in the WT model during all our simulation time, indicating its importance to the structural arrangement. We then calculated the intermolecular interaction potential (long-range Coulombic interaction energy + short-range Lennard-Jones energy) between these residues for the WT complex and for the R87C complex. The average for residues R87 and E624 was −161 kJ/mol ± 0.21 kJ/mol (−142.88 ± 16 kJ/mol in the replicate), while for C87 and E624 it was −1.29 ± 0.38 kJ/mol (−0.3 ± 0.14 kJ/mol in the replicate). There was also a decrease for residue E689, where the average intermolecular interaction potential for residues R87 and E689 was −167 ± 0.37 kJ/mol (−166.47 ± 0.38 kJ/mol in the replicate), while for C87 and E689 it was −0.26 ± 0.06 kJ/mol (−0.36 ± 0.03 kJ/mol in the replicate). This indicates that the interaction between residues 87, E624, and E689 is much more attractive in the WT complex than in the R87C complex.

In order to compare the CYFIP2 WT and the R87C isolated structures with the complex structures, the same analyses were conducted during the CYFIP2 WT and R87C simulations (Figure S5A–F). In the CYFIP2 WT simulation, residue 87 made about 100 contacts with residue E624 at the beginning and the end of the simulation, with a minimum distance of about 0.2 nm. Although, from 20 to 60 ns of simulation, the number of contacts between them decreased to almost 0, and the distance increased to 5 nm, showing that these two residues were apart in solution, which may be due to the loop flexibility. On the other hand, these two residues had 0 contacts along the entire simulation, with around 0.9 nm between them in the R87C simulation. This implies that the mutation reduced the contacts between these two residues and that, once again, these contacts were not accommodated in our simulation time. The same happened for the interaction between the residues 87 and E689. In the WT simulation, there were around 50 contacts between them until 60 ns of simulation, with 0.5 nm between them. However, there were no contacts between these residues along the entire R87C simulation, which implies that the mutation also reduces the interaction between these residues. The same behavior was already observed within the complex. This could imply that the interactions between these residues are present in isolated CYFIP2, but they are in some way stabilized inside the complex, becoming even more relevant for the proper complex structure.

Moreover, we evaluated the interactions of residue 87 with residue Y151 of WAVE1. Regarding these residues, we could see differences between the complexes and that the number of contacts varied more during the simulation. For the WT complex, the number of contacts varied between 150 and 250, with a minimum distance between 0.2 and 0.3 nm. For the R87C complex, the number of contacts varied between 50 and 150, with a minimum distance between 0.3 and 0.4 nm. An indication that, although there was a loss of contacts with the WAVE1 protein, the loss of internal contacts between CYFIP2 residues, causing the destabilization of the loop, seemed to be more relevant in our simulation time.

## 3. Discussion

Our molecular dynamics simulations demonstrated a movement of the loop where residue 87 of the protein resides. In the structure of both the isolated R87C CYFIP2 and the R87C complex, this loop was distant from the rest of the protein when compared with the WT complex and with the crystallographic X-ray structure of the WRC complex [13]. Through PCA, we also noticed a greater movement of this loop in the R87C complex. The displacement of the loop in CYFIP2 R87C showed the flexibility of this region, which could impair the fit and interaction between WAVE1 and CYFIP2 (Figure 2).

In the first studies to understand the modulation of the WRC complex, the consensus was that the complex disaggregates when interacting with Rac1-GTP and that this allowed the interaction of WAVE with the Arp2/3 complex [10]. The revised and current model of WRC activation points out that this complex does not disaggregate. It undergoes a remodeling that allows the interaction of the WAVE’s VCA domain with Arp2/3 [8,24]. In a recent preprint paper, Ding and colleagues [23] brought a description of the WRC activation mechanism by the interaction of RAC1 in the “A site”. The interaction of Rac1 promotes a structural rearrangement that directly affects the loop within R87 and its interaction with WAVE1, causing the activation. Therefore, the structural alteration due to the R87C variant observed in our simulations could significantly impact the complex remodeling and its regulation. Moreover, through co-immunoprecipitation analyses, it has already been pointed out that the CYFIP2 R87C variant does not have reduced interaction with WAVE1, but the specific interaction with the WAVE’s VCA domain is impaired [7]. It has also been shown that the variant leads to the constant activation of the complex, even without its activation by RAC1 [18].

As already noted in this and other studies [7,17], residue R87 forms hydrogen bonds with E624 and E689. These crucial interactions were lost in the CYFIP2 R87C models, and the absence remained throughout our simulations (Figure 6, Figure 7 and Figure S4). Arginine residues have a long side chain and are positively charged at physiological pH, including in hydrophobic environments inside proteins [25]. These characteristics make them residues prone to strong and stable interactions with polar residues, mainly in the interaction with the negatively charged carboxyl groups of glutamate and aspartate side chains. In this work, we point out that this loss of interaction, with the displacement of the interaction of E624 and E689 with R87, could destabilize the configuration of the 80–110 loop, which seems to have an essential role in the anchoring of the WAVE1 protein within the complex, as shown by Ding and colleagues [23] in the process of the WRC activation by the Rac1 interaction. Moreover, this destabilization could lead to the WRC activation caused by the mutation, as in some of our simulations we started to see Y151 moving from its initial pocket.

This disruption of the 80–110 loop structure could also impact CYFIP2 stability and its interaction with other proteins. Despite being more studied in the context of the WRC complex, CYFIP2 is also known for its interaction with other complexes and seems to participate in different cellular processes. Initially, CYFIP2 was identified and named PIR121 due to the induction of its gene by p53 [26]. It was later renamed for the identification of its interaction with the FMRP protein, an RNA-binding protein (RBP) involved in translation regulation [27]. An analysis of the CYFIP2 interactome showed interactions with several proteins besides other WRC components, including RBPs and proteins related to stress granule formation [20]. HeLa cells expressing R87 variants of CYFIP2 showed the formation of aggregates containing CYFIP2 within them. Interestingly, these aggregates co-localized with Ago2, one of the RBPs found in the CYFIP2 interactome [20]. Molecular dynamics simulations of CYFIP1 have already indicated its character as a “moonlighting” protein [28]. This type of protein has different functions depending on its location or conformation. For CYFIP1, when the protein adopts a more globular conformation, it favors its interaction with the FMRP complex. However, when it adopts a planar conformation, its interaction with the WRC complex is favored [28]. Due to its high similarity, CYFIP2 is very likely to demonstrate the same behavior. A small structural change, such as that of the 80–110 loop, could also impact the regulation of this conformational transition.

Therefore, the loop destabilization can impact protein function, not just in the WRC complex. However, this is difficult to verify by computer modeling because little is known about the exact interaction regions of CYFIP2 and these other proteins. In addition, experimental evidence shows a trend towards lower stability of CYFIP2 R87C but without conclusive results [29]. In vitro studies are still needed to better understand the impacts of this mutation outside the context of the WRC complex and the impacts on CYFIP2 stability.

This work brings new in silico evidence on the possible structural alterations of the CYFIP2 protein resulting from the R87C modification, also present in patients with EIEE. This evidence seems to agree with the hypothesis of weakening binding with WAVE and with experimental results regarding the interaction of CYFIP2 R87C and WAVE1. Thus, through our simulations, we demonstrate the possible mechanism of structural modification associated with the pathological effects of the CYFIP2 R87C variant. The description of this behavior may guide further studies in the understanding of the molecular pathways and mechanisms compromised by CYFIP2 mutations. In addition, it could guide research in the development of a CYFIP2-targeted therapy.

## 4. Materials and Methods

### 4.1. Tridimensional Models

The alignment of the amino acid sequences of CYFIP2 and CYFIP1 was performed in clustal Ω [30], and the proteins share 88% identity. Due to high similarity, the alignment of the amino acid sequence of CYFIP2 and the X-ray structure of the CYFIP1 in WRC (PDB 3P8C) was performed in Hhpred [31] and used as an input for Modeller version 10.0 [32]. The homology modeling was carried out using the automodel script [32] with the structure PDB 3P8C as a template to construct the CYFIP2 and CYFIP2 R87C models. In the same way, the alignment of the amino acid sequence of CYFIP2-NCKAP1-WAVE1 was used for the construction of the WRC models. The resolved WRC complex includes the structures of CYFIP1, NCKAP1, WAVE1, Abi2, and BRK1. The structures of Abi2 and BRK1 were excluded from the models. The WAVE1 protein is partially solved; therefore, its region between residues 186 and 485 was substituted by a linker (GlyGlySer)6 (the same used by Chen et al. [13] for solving the X-ray structure). Other unsolved regions were small and were modeled using the Modeller loop-refinement algorithm. The residues were renumbered sequentially according to Table 1.

The best model out of 100 generated tridimensional models was chosen based on the DOPE score [33]. The complex with CYFIP2 R87C was constructed in the same way as described above. All models were validated using Molprobity [34] geometric evaluations and the Ramachandran plot. Here, the WRC structure, including the CYFIP2 sequence, is denoted as complex WT, and the structure including the CYFIP2 R87C sequence is denoted complex R87C.

### 4.2. Molecular Dynamics of CYFIP2 WT and CYFIP2 R87C

The molecular dynamics (MD) of isolated CYFIP2 WT and CYFIP2 R87C were performed using GROMACS 2020.4 [35] using the CHARMM36m [36] force field to describe the atomic interactions. The initial simulation structure of each system was obtained from the homology modeling mentioned in the previous section. The atomic coordinates of both structures were submitted to the H++ server [37], then were placed in a solvated dodecahedron box filled with TIP3P [38] water molecules. The distance between protein edges and box edges was set to 2 nm in all axes. The protein binding lengths were controlled using the LINCS [39,40] algorithm, and those of water were controlled using the SETTLE [41] algorithm. The interactions between unbound atoms, van der Waals and Coulomb, were evaluated with a cutoff radius of 1.3 nm, and corrections for electrostatic interactions were calculated using the particle mesh Ewald method (PME) [42].

The first step of energy minimization was performed using the steepest descent integrator [43] at 10,000 steps and an integration step of 2 fs with the atomic coordinates of the protein restrained to allow better a adjustment of the solvent around the protein. The energy system minimization remained through short NVT and NPT ensemble simulations at 310 K. The minimization began in NVT with 26 ps MD (time steps of 0.5 and 1 fs), keeping the atomic coordinates of CYFIP2 WT and CYFIP2 R87C restrained in each system. Then, the minimization continued in NPT with increasing integration times of 0.5, 1, and 2 fs, for a total time of 32 ps with the protein still restrained, followed by 42 ps (time step of 2 fs) with the protein free. The temperature was controlled by the V-rescale thermostat [44] with the protein, solvent, and ions separately coupled and a coupling constant of 0.2 ps. Likewise, the pressure of the systems was controlled by a Berendsen barostat [45] with a constant of 0.2 ps in 1 bar. The trajectory acquisition was performed in an NPT ensemble for 100 ns with a time step of 2 fs. The coordinates of MD were collected every 100 ps.

### 4.3. Molecular Dynamics of the WRC Complex

After analyzing the structures obtained in isolated CYFIP2 simulations, we performed the complex simulations to investigate if the same pattern would be observed, even with the interaction between CYFIP2 and WAVE1. The simulation of CYFIP2 within the WRC complex was performed with GROMACS 2020.4 [35] and a CHARMM36m (version February 2021) [36] force field. Protonation states were assigned using H++ server version 3.2 [37], considering the pH of 7.4. The models were immersed in a dodecahedron simulative box that was 15 Å away from the protein surface, filled with TIP3P [38] solvent molecules, and rendered electroneutral by the introduction of chlorine/sodium counterions (the systems’ complete atomic compositions are shown in Table 2).

The minimization using the steepest descent algorithm [43] was performed with a number of steps where the maximum force was <1000.0 kJ/mol/nm to regularize the structures. The optimization and relaxation of the solvent and ions were performed in two steps (NVT and NPT) by simulating the system at a temperature of 310 K and a pressure of 1 bar. The models were simulated using GROMACS 2020.4 [35] for 50 ns. The electrostatic interactions were treated using the particle mesh Ewald (PME) algorithm [42] with a 1.2 nm cutoff. The simulation was carried out with a time step of 2.0 fs at a constant temperature of 310 K using the modified Berendsen thermostat (V-rescale algorithm) [44] and a pressure of 1 bar using the Parrinello–Rahman method [46] for pressure coupling. Coordinate files were saved every 10 picoseconds (ps). The simulations were performed in duplicates.

### 4.4. Molecular Dynamics Analysis

The resultant trajectory of all simulations was processed using the gmx trjconv. The root-mean-square deviations (RMSD), root-mean-square fluctuations (RMSF), solvent-accessible surface areas (SASA), gyration radius (Rg), number of contacts, minimum distance between residues, and principal component analysis (PCA) were built using the GROMACS 2020.4 package [35]. The trajectories were visualized using VMD version 1.9.3 [47], and the images were obtained with UCSF Chimera version 1.14 developed by the Resource for Biocomputing, Visualization, and Informatics at the University of California, San Francisco [48]. All graphs were plotted with qtgrace.

The structure’s conformations were clustered with the g_cluster program in GROMACS 2020.4 using the Gromos algorithm [49]. In order to obtain a maximum number of fewer than 20 clusters from the 5000 frames, a threshold value of 0.3 nm was used. The centroid structure of the most populated cluster from each simulation was used for comparing interactions.

## Figures and Tables

**Table 1:** Original residue notation compared to notation in our models.

Protein	Original Residue Numbering	Residue Numbering in Models
CYFIP2	1–1252	1–1252
NCKAP1	1–1127	1253–2379
WAVE1	WAVE1(1–186)-(GlyGlySer)6-(485–559)	WAVE1(2380–2544)-(GlyGlySer)6-(2563–2622)

**Table 2:** The atomic compositions of all simulated systems.

System	Composition (Residues/Molecules)						
	Chain A (CYFIP2)	Chain B (NCKAP1)	Chain C (WAVE1)	Na+	Cl−	Water	Total
Complex WT	1252	1127	242	21	-	398,728	401,371
Complex R87C	1252	1127	242	22	-	372,980	375,624

## Data Availability

Not applicable.

## Supplementary Materials

The supporting information can be downloaded at: https://www.mdpi.com/article/10.3390/ijms23158708/s1.
