# Supplementary material for: Molecular Dynamics of CYFIP2 Protein and Its R87C Variant Related to Early Infantile Epileptic Encephalopathy
Source: Int J Mol Sci. 2022 Aug 5;23(15):8708. doi: 10.3390/ijms23158708 (PMC9368851; doi:10.3390/ijms23158708)
Supplement: Supplementary file 1 [file ijms-23-08708-s001.zip › ijms-1765190-supplementary.pdf]

International Journal of *Molecular Sciences*

Article

Molecular Dynamics of CYFIP2 protein and its R87C variant related to Early Infantile Epileptic Encephalopathy

Ísis V. Biembengut [1], Patrícia Shigunov [2], Natalia F. Frota [3,4], Marcos R. Lourenzoni [4], Tatiana A. C. B. de Souza [1,*]

[1] Structural and Computational Proteomics Laboratory, Carlos Chagas Institute, FIOCRUZ-PR, Curitiba/PR, 80320-290, Brazil
[2] Laboratory of Basic Biology of Stem Cells, Carlos Chagas Institute, FIOCRUZ-PR, Curitiba/PR, 80320-290, Brazil
[3] Federal University of Ceara (UFC), Campus do Pici (Bloco 873), Fortaleza/CE, 60440-970, Brazil
[4] Research Group on Protein Engineering and Health Solutions (GEPeSS), Fundação Oswaldo Cruz Ceará (Fiocruz-CE), São José, Precabura, Eusébio/CE, 61760000, Brazil
* Correspondence: tatiana.brasil@fiocruz.br

1. Supporting Information

Table S1. Model geometry evaluated by Molprobity

Parameter	Model complex WT	Model complex R87C
Poor rotameters	1.94%	2.19%
Favored rotameters	92.71%	92.96%
Ramachandran outliers	0.38%	0.5%
Ramachandran favored	97.44%	97.55%
Bad bonds	0	0
Bad angles	1.35%	1.26%

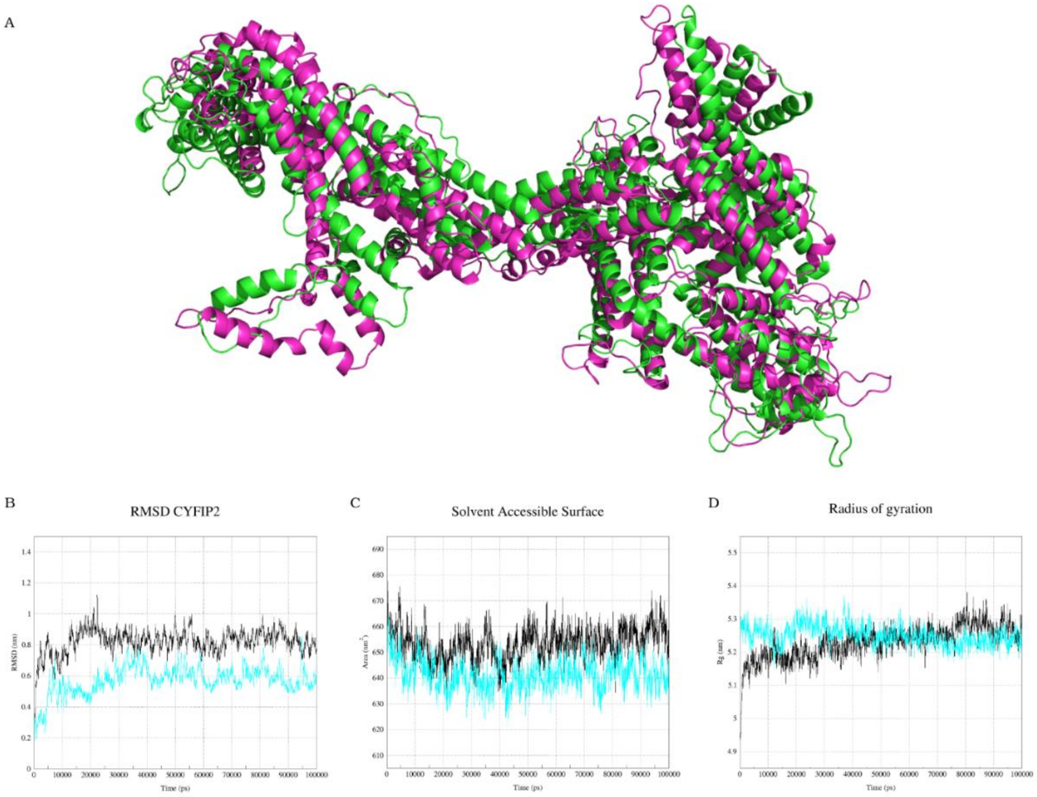

Figure S1. Structure and conformational evolution through the entire MD simulation of CYFIP2 WT and CYFIP2 R87C models. Ribbon representation of CYFIP2 WT (green) and CYFIP2 R87C (pink) (A). Plot of Root Square Mean Deviation (RMSD) calculated using the original model as a reference for the backbone of CYFIP2 (B), Plot of solvent-accessible surface area (SASA) (C). Plot of the radius of gyration (Rg) (D). Data for the WT simulation is represented gray lines. Data for R87C simulation is represented cyan lines.

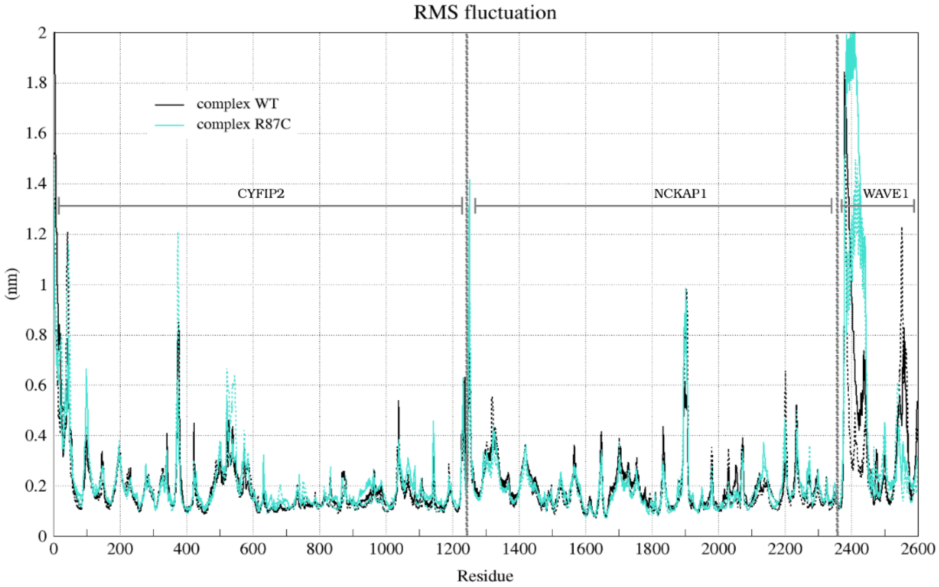

Figure S2. Root-mean-square fluctuation (calculated with reference from average structure fitting of C-alphas for the entire structure through the entire trajectory) for each residue. Data for complex

WT simulation is represented by dark (first replica) and light (second replica) gray lines. Data for complex R87C simulation is represented by dark (first replica) and light (second replica) cyan lines.

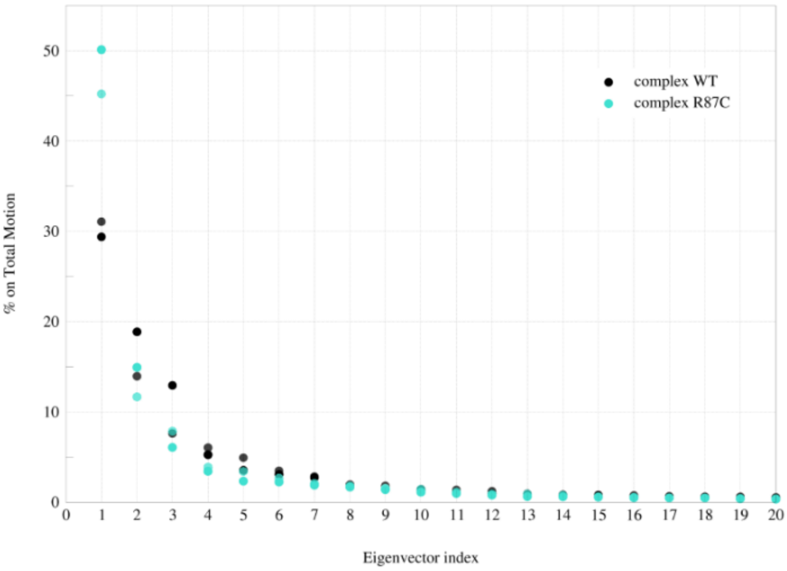

Figure S3. Percentage of the eigenvalues in the principal component analysis.

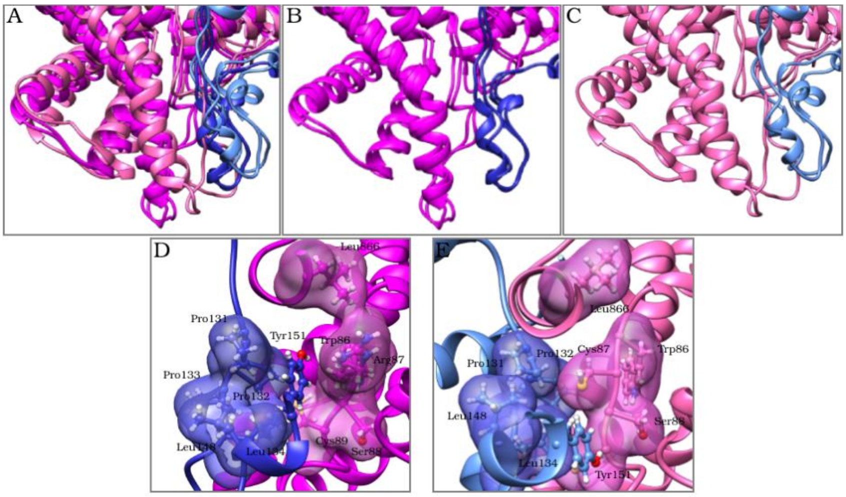

Figure S4. Detail of residues 80-120 from CYFIP2 and residues 131-151 from WAVE1 in superimposed structures of the first cluster centroid for both replicas from WT complex (darker colors) and R87C complex (lighter colors) (A). Representation of CYFIP2 in magenta and WAVE1 in blue. Superimposed models (A), only WT models (B), and only R87C models (C). Visualization of residues that form a pocket around Y151 from WAVE in complex WT (D), and in complex R87C (E). In D and E, the structures are from the first cluster centroid from the first replicate.

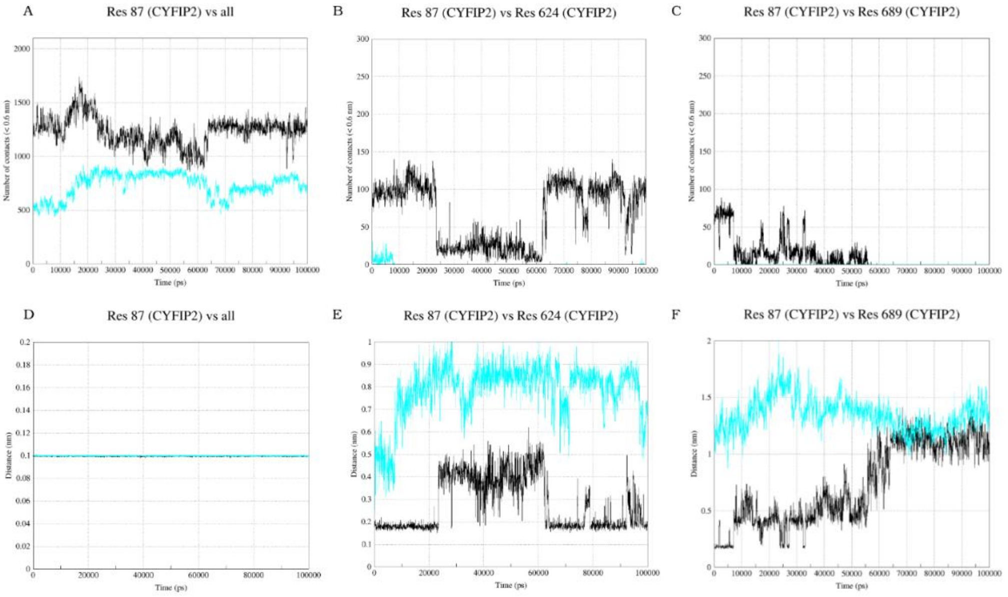

Figure S5. Number of contacts between residue R/C87 and all other residues (A), E624 CYFIP2 (B), and E689 CYFIP2 as a function of simulation time (ps). Minimal distance between residue R/C87 and all other residues (E), E624 CYFIP2 (F), and E689 CYFIP2 (G) as a function of simulation time (ps). Data for complex WT simulation is represented by dark (first replica) and light (second replica) gray lines. Data for complex R87C simulation is represented by dark (first replica) and light (second replica) cyan lines.
